# Case Report: Gastrointestinal neuroendocrine carcinoma with SMARCA4 deficiency: a clinicopathological report of two rare cases

**DOI:** 10.3389/fonc.2023.1290717

**Published:** 2023-11-28

**Authors:** Ping Zhou, Yiyun Fu, Weiya Wang

**Affiliations:** Department of Pathology, West China Hospital, Sichuan University, Chengdu, China

**Keywords:** SWI/SNF, SMARCA4, BRG1, gastric cancer, neuroendocrine carcinoma

## Abstract

**Background:**

Gastrointestinal neuroendocrine carcinoma (GI NEC) is a rare but highly malignant neoplasm with an aggressive clinical course. SMARCA4 is one of the subunits of the SWI/SNF chromatin remodeling complex. SMARCA4 deficiency can occur rarely in subsets of NECs. Reports of the clinicopathological features of GI NECs with SMARCA4 deficiency are limited.

**Methods:**

In this study, we retrospectively reported two rare cases of GI NEC with SMARCA4 deficiency and described the clinicopathological, radiographic and histopathological features.

**Results:**

Case 1 was a 43-year-old male with a stage cT3NxM1, IV tumor. Case 2 was a 64-year-old female with a stage cT4aN1M0, IIIA tumor. Both tumors presented as ulcerated masses with infiltration. Pathological examination indicated a solid architecture with poorly differentiated morphology, and complete loss of SMARCA4 (BRG1) was found. Immunohistochemical staining showed positivity for Syn, CgA and CD56. The Ki-67 index was 90% and 70%, respectively. None of the cases had mismatch repair (MMR) deficiency. Case 1 received treatment with chemotherapy and anti-PD-1 immunotherapy. He did not respond to treatment, and died 9 months later. Case 2 received neoadjuvant chemotherapy before surgical treatment, and the tumor showed TRG3 in response to neoadjuvant chemotherapy, chemotherapy and anti-PD-1 immunotherapy were continued after surgical resection. There was no evidence of disease for 10 months.

**Conclusions:**

GI NEC with SMARCA4 deficiency is a rare entity of gastric NEC. SMARCA4 may be a promising targetable and prognostic biomarker. BRG1 immunohistochemical staining could be performed for GI NECs. Further studies with a larger cohort will be needed.

## Introduction

Gastric cancer (GC) is one of the most common malignancies globally and has poor outcomes, especially in Asia (1, 2). Neuroendocrine neoplasms are epithelial neoplasms with neuroendocrine differentiation. Gastrointestinal neuroendocrine carcinoma (GI NEC) is a rare but highly malignant neoplasm with an aggressive clinical course (3). Chemotherapy has been the mainstay of treatment in unresectable or advanced high-grade GI NEC (4). A randomized clinical trial demonstrated that both etoposide plus cisplatin (EP) and irinotecan plus cisplatin (IP) can be standard first-line chemotherapy options for advanced neuroendocrine carcinoma (NEC) (4).

Recently, switch/sucrose non-fermentable (SWI/SNF) complexes were found to be a highly preserved group of multiprotein complexes that regulate chromatin remodeling and play an important role in proliferation, differentiation and tumor suppression (5, 6). *SMARCA4*, a tumor suppressor gene and one of the subunits of the SWI/SNF chromatin remodeling complex, encodes Brahma-related gene 1 (BRG1) (6). *SMARCA4* mutations were found to be present in a diverse set of cancer types at frequencies of up to 16% in solid tumors from 131,668 cancer patients (7). SMARCA4/BRG1 deficiency has been detected in a wide variety of tumors (8–15), such as small cell carcinoma of the ovary, hypercalcemic type (SCCOHT) and thoracic SMARCA4 undifferentiated tumors.

*SMARCA4* mutations have been reported in a few neuroendocrine carcinomas. Germline and somatic alterations in the *SMARCA4* gene and loss of BRG1 protein expression have been established as defining events in small cell carcinoma of the ovary, hypercalcemic type (SCCOHT) (8). SMARCA4 deficiency can be present in TTF-1-negative neuroendocrine carcinomas (16). GI NEC with SMARCA4 deficiency may represent a rare phenotype of GI NEC and has not been reported in published English literature. Herein, we report two rare cases of GI NEC with SMARCA4 deficiency and provide insight into the clinicopathological features of this highly aggressive malignant tumor.

## Methods and patients

### Patient collection

Data from two cases of GI NEC with SMARCA4 deficiency were reviewed between January 2020 and December 2022 from the database of the Department of Pathology, West China Hospital, Sichuan University. Clinical and radiographic features were obtained from patients’ medical records and follow-up. Ethics approval was obtained from the respective ethics committees of West China Hospital, Sichuan University, China (NO.2022317).

### H&E and immunohistochemical staining

H&E and immunohistochemical staining was performed on 4-μm-thick unstained sections of representative formalin-fixed paraffin-embedded blocks. Immunohistochemistry was performed with the EnVision detection system. Antigen retrieval and staining were performed using standardized automated protocols in the presence of appropriate controls. Staining for SMARCA4 (anti-BRG1 antibody, 1:200 dilution, clone EPNCIR111A; Abcam, Cambridge, MA) was performed, as well as pancytokeratin (PCK) (clone AE1/AE3, ZSGB-BIO), epithelial membrane antigen (EMA) (clone GP1.4, ZSGB-BIO), CK20 (clone EP23, ZSGB-BIO), CK7 (clone EP16, ZSGB-BIO), CK8/18 (clone 5D3, MXB), p53 (clone D0-7, ZSGB-BIO), RB (clone 13A10, CELNOVTE), synaptophysin (Syn) (clone EP158, ZSGB-BIO), chromogranin A (CgA) (clone LK2H10, ZSGB-BIO), CD56 (clone UMAB83, ZSGB-BIO), Ki67 (clone MIB-1, ZSGB-BIO), LCA (clone 2B11&PD7/26, ZSGB-BIO), MLH1 (clone ES05, ZSGB-BIO), MSH2 (clone RED2, ZSGB-BIO), MSH6 (clone EP49, ZSGB-BIO) and PMS2 (clone EP51, ZSGB-BIO). Staining results were determined by 2 independent pathologists.

### In situ hybridization of Epstein−Barr virus-encoded small RNA (EBER)

We stained 4-μm-thick sections for *in situ* hybridization to examine the Epstein−Barr virus (EBV) infection status. The EBER probe was detected using the PNA ISH Detection Kit (Dako).

## Results

### Clinical presentation

Detailed clinical features of the two patients of GI NEC with SMARCA4 deficiency are summarized in **Table 1**.

**Table 1:** Clinical features of gastrointestinal NEC with SMARCA4-deficiency.

Cases	Age/ Gender	Symptoms	Tumor size	Tumor location	TNM Stage	Treatment	MTS/Survival
Case 1	43/M	Epigastric pain with yellowness of the skin for seven months	1.5 cm	Duodenal papillae	cT3NxM1, IV	Chemotherapy (etoposide plus cisplatin) and anti-PD-1 immunotherapy (serplulimab)	DOD (9 months), liver and intraperitoneal MTS, and retroperitoneal lymph node MTS.
Case 2	64/F	Epigastric pain for nine months	2.9 cm	The greater curvature of the stomach	cT4aN1M0, IIIA	Surgical resection followed by chemotherapy (etoposide plus cisplatin), and continued to be treated with chemotherapy (cisplatin) and anti-PD-1 immunotherapy (sintilimab)	NED (10 months)

M: male; F: female; y, years; TNM, tumor-node-metastasis; PD-1, programmed cell death protein 1; NED, no evidence of disease; DOD, died of disease, MTS, metastasis.

### Case 1

**Figure 1:**
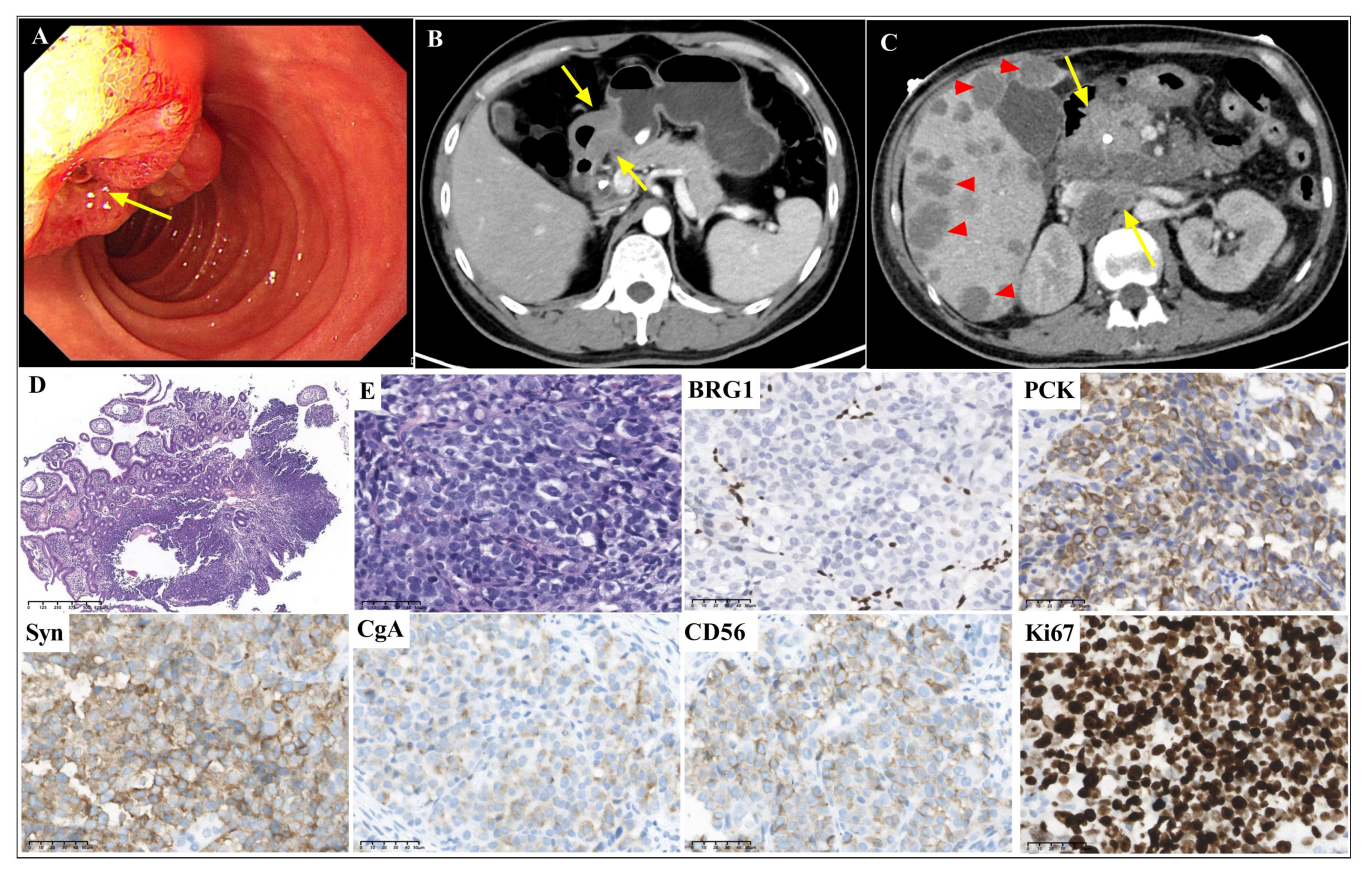
Gastroscopic, radiological, histopathological and immunohistochemical features of case 1 Gastroscopy (**A**, arrow) and abdominal CT scans (**B**, arrow) showed a thickened lesion in duodenal papilla. The abdominal CT scan showed multiple liver metastases (**C**, triangles), intraperitoneal metastases and retroperitoneal lymph node metastases (**C**, arrows) after chemotherapy. The biopsy showed a solid pattern with poorly differentiated tumor cells with inflammatory infiltration (**D**, magnification x40; and **E**, magnification x400). BRG1 was deficient. PCK, CgA, Syn and CD56 were positively stained. The Ki67 index was approximately 90% (magnification x400).

A 43-year-old male patient presented with epigastric pain with yellowness of the skin for seven months. Gastroscopy showed a 1.5-cm thickened lesion in the duodenal papillae (**Figure 1A**), and a biopsy was performed. A computed tomography (CT) scan of the abdomen showed a 1.5-cm thickened lesion in the duodenal papillae (**Figure 1B**) and multiple nodules in the liver, indicating metastases of the liver. The clinical stage was cT3NxM1, IV.

Morphological analysis of the biopsy tissue showed poorly differentiated cells forming a solid architecture of neuroendocrine morphology (**Figures 1D, E**). The large-sized tumor cells were epithelioid ovoid with abundant cytoplasm. Nuclei were round and pleomorphic. Necrosis was not found in the small biopsy. Lymphocyte, eosinophil, and neutrophil infiltration was observed. Immunohistochemistry (**Figure 1**) indicated complete loss of BRG1 in the tumor nuclei, with endothelial and inflammatory cells as internal positive controls. CgA, Syn and CD56 were positively stained. The Ki67 index was approximately 90%. The tumor was positive for epithelial markers (PCK, CK8/18 and EMA) and negative for LCA. The expression of INI1, MLH1, MSH2, MSH6 and PMS2 was retained. *In situ* hybridization for EBER was negative. Due to the small biopsy, there was neither abundant tumor tissue nor sufficient well-preserved nucleic acids for next-generation sequencing (NGS) after immunohistochemistry.

Based on the morphological and immunohistochemical features, the tumor was diagnosed as GI NEC with SMARCA4 deficiency. The patient received chemotherapy (etoposide plus cisplatin) and anti-programmed cell death protein 1 (anti-PD-1) immunotherapy (serplulimab) for three cycles. The CT scan of the abdomen and gastroscopy showed a 3.0-cm thickened lesion in the duodenal papillae, multiple liver metastases (**Figure 1C**, triangles), and intraperitoneal metastases, and enlarged and partially fused retroperitoneal lymph nodes (**Figure 1C**, arrows). The tumor did not respond to treatment with chemotherapy and anti-PD-1 immunotherapy, and the patient died 9 months later.

### Case 2

**Figure 2:**
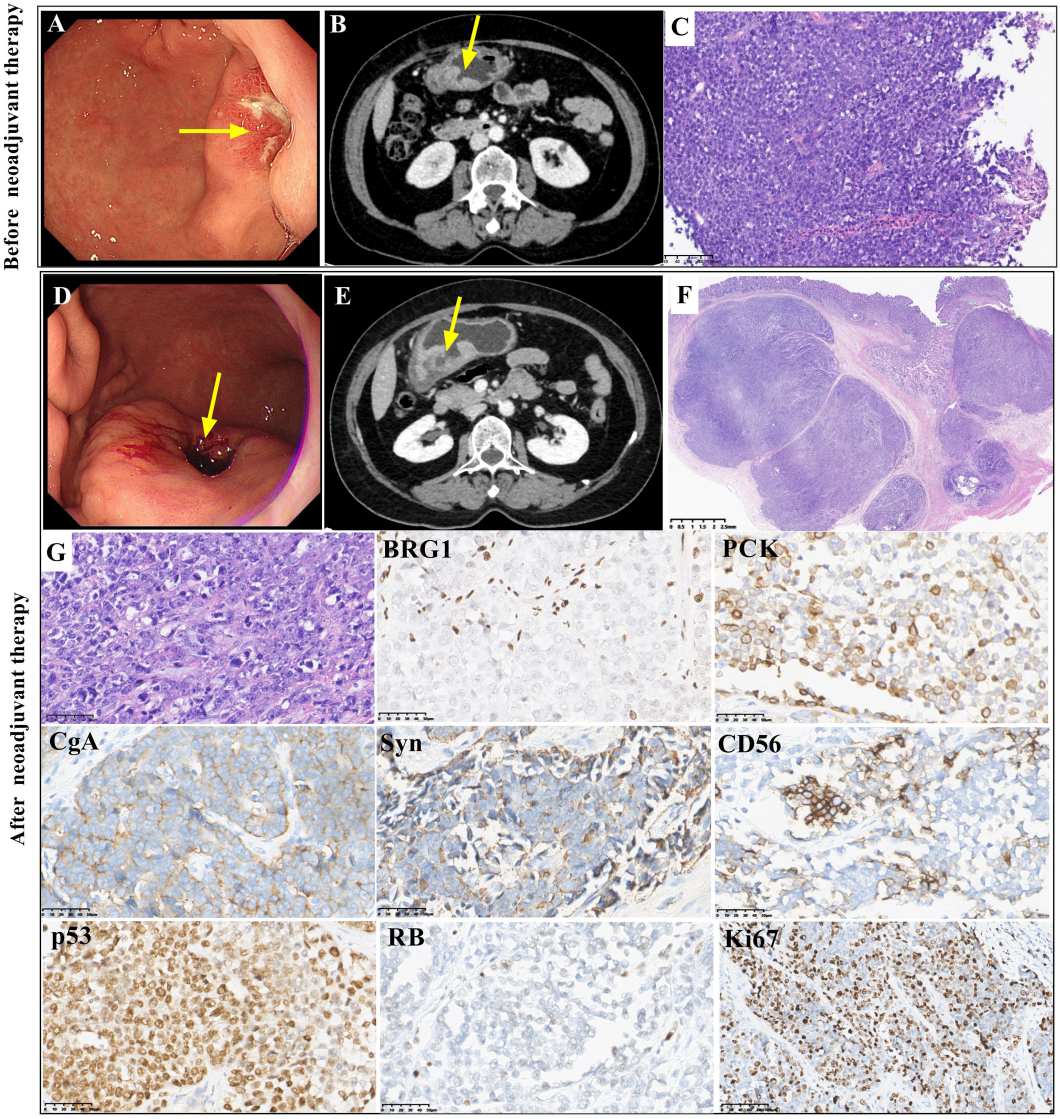
Gastroscopic, radiological, histopathological and immunohistochemical features of case 2 Gastroscopy (**A**, arrow) and abdominal CT scans (**B**, arrow) showed an irregularly thickened area in the greater curvature of the stomach before neoadjuvant treatment. The biopsy showed a solid pattern with poorly differentiated tumor cells before neoadjuvant treatment (**C**, magnification x100). Gastroscopy (**D**, arrow) and abdominal CT scans (**E**, arrow) showed a larger irregular thickened area of the greater curvature of the stomach after neoadjuvant treatment. The resected tumor showed a solid pattern with poorly differentiated tumor cells after neoadjuvant treatment, and there was no obvious response to neoadjuvant therapy (TRG3) (**F** and **G**, magnification x400). Both biopsy tissue and surgical resected tumor showed similar immunohistochemical staining. BRG1 was deficient. PCK staining was positive. CgA, Syn and CD56 were positively stained. P53 staining was positive, and RB staining was deficient. The Ki67 index was approximately 70% (magnification x400).

A 64-year-old female patient presented with epigastric pain for nine months. Gastroscopy (**Figure 2A**) and a CT scan of the abdomen (**Figure 2B**) showed a 2.9-cm irregular ulcerative tumor in the greater curvature of the stomach, and a biopsy was performed. The clinical stage was cT4aN1M0, IIIA. The patient was administered neoadjuvant treatment with chemotherapy (etoposide plus cisplatin) for two cycles. Gastroscopy (**Figure 2D**) and a CT scan of the abdomen (**Figure 2E**) showed a larger irregular ulcerative tumor in the greater curvature of the stomach after neoadjuvant chemotherapy. The clinical assessment was progressive disease (PD). Then, surgical resection was performed. On gross examination, there was an ulcerative tumor in the greater curvature of the stomach measuring 2.6 cm×2.5 cmx1.1 cm.

The biopsy tissue and resected tumor showed poorly differentiated morphology with solid architecture (**Figures 2C, F, G**). Round, pleomorphic nuclei with prominent nucleoli were large and irregular. Mitoses were frequent. The tumor cells in the resected specimen invaded the muscularis propria. Vascular invasion was observed. The pathological stage was yT2N2M0, IIB. Few lymphocytes and neutrophils were present. The tumor regression grade (TRG) was TRG3 (without an obvious response to neoadjuvant treatment) (**Figure 2F**). The immunohistochemical staining results of the resected tumor were similar to those of the biopsy. Complete loss of BRG1 was observed in the tumor nuclei, which was similar to the result for case 1 (**Figure 2**). Immunohistochemistry showed positivity for PCK. CgA, Syn and CD56 were positively stained. P53 immunoreactivity was positive. Complete loss of RB was found. The Ki67 index was approximately 70%. Immunohistochemical staining for CK7, CDX2, CK20, HER2 and SSTR2 showed negative results. The expression of INI1, ATRX, MLH1, MSH2, MSH6 and PMS2 was retained. *In situ* hybridization for EBER was negative. Regrettably, the patient rejected NGS detection of her sample.

Based on the morphological and immunohistochemical features, the tumor was diagnosed as GI NEC with SMARCA4 deficiency. The patient continued to be treated with chemotherapy (cisplatin) and anti-PD-1 immunotherapy (sintilimab) after surgery. There was no evidence of disease for 10 months.

## Discussion

Kadoch et al. demonstrated that approximately 20% of all human cancers harbor mutations in SWI/SNF chromatin-remodeling complexes (17). *SMARCA4*, one of the subunits of the SWI/SNF chromatin remodeling complex, is a tumor suppressor (6). *SMARCA4* mutations occurred in 8% (20/258) of gastric cancers in a TCGA analysis and 10% (5/50) of gastric cancers in Takeshima’s study (1, 18). Loss of SMARCA4 is associated with adverse clinical characteristics (19, 20). SMARCA4 (BRG1) deficiency occurs rarely in subsets of NECs, such as SCCOHT (8) and lung neuroendocrine carcinomas (16). The present report describes two rare patients diagnosed with GI NEC with SMARCA4 deficiency, aged 43 and 64 years, who presented with ulcerated and transmural masses with infiltration and were staged as cT3NxM1, IV in case 1 and cT4aN1M0, IIIA in case 2 at the time of diagnosis.

Inactivation of SMARCA4 is more likely to occur in gastric cancer with a solid and undifferentiated morphology, presenting in large and locally advanced tumors (21–23). Aberrant SMARCA4 protein expression was reported to be frequently observed in 49% (25/51) of solid-type poorly differentiated adenocarcinomas and nonsolid-type poorly differentiated adenocarcinomas (7.5%, 3/40) (20). However, GI NEC with SMARCA4 deficiency has not been reported in the published English literature. We reported two rare GI NECs that presented with a solid architecture and poorly differentiated morphology and showed complete loss of BRG1 expression. BRG1 immunohistochemical staining is useful for identifying SMARCA4-deficient tumors. In routine practice, screening BRG1 expression could be performed for GI NEC in pathological diagnosis.

A group of tumors with similar morphological features should be excluded before diagnosing GI NEC with SMARCA4 deficiency. The differential diagnosis includes gastric carcinoma with SMARCA4 deficiency, undifferentiated carcinomas, EBV-associated carcinoma, lymphoma, melanoma, germ cell neoplasms, and so on. GI NEC often diffusely expresses neuroendocrine markers, including chromogranin, synaptophysin and CD56, and tumor cells express epithelial markers. Gastric carcinoma with SMARCA4 deficiency can be distinguished by gland architecture of differentiation. Some undifferentiated tumors with a neuroendocrine-like phenotype may show variable positivity for synaptophysin, but neither of the cases expressed more than one neuroendocrine marker. Decreased expression of PCK was observed in 58.6% (17/29) of gastric SMARCA4-deficient undifferentiated carcinomas (21). Staining for LCA, CD138, CD38, MUM1, and anaplastic lymphoma kinase (ALK) is helpful for diagnosing lymphopoietic system tumors, including plasmablastic lymphoma and ALK-positive large B-cell lymphoma. HMB45, S100, and MART-1 can be helpful for diagnosing melanoma. A combination of antibodies, including Sal-like transcription factor 4 (SALL4), octamer-binding transcription factor 3/4 (OCT3/4) and α-fetoprotein (AFP), have been used to diagnose germ cell tumors.

Poorly differentiated neuroendocrine carcinoma of the digestive system has a dismal prognosis with limited treatment options. Systemic platinum-based treatment is the standard treatment for GI NEC. For high-grade NEC in the GI tract, multiagent chemotherapy was found to be associated with superior survival compared with single-agent chemotherapy, which was superior to no chemotherapy (3). programmed cell death receptor 1/ programmed cell death ligand 1 (PD-1/PD-L1) expression is a frequent occurrence in poorly differentiated neuroendocrine carcinomas of the digestive system. Checkpoint blockade targeting the PD-1/PD-L1 pathway may have a potential role in treatment (24, 25). There are no published studies on a recommended treatment for GI NEC with SMARCA4 deficiency. Patients with SMARCA4-altered GC do not benefit from chemotherapy in stages II and III (P=0.623 and 0.678) (26). SMARCA4 alteration in GC remains a significant unfavorable prognostic factor (median survival 14 versus 26 months, p=0.002) in patients with stage III disease who receive chemotherapy (26). Two patients with tumors localized to the gastroesophageal junction received neoadjuvant chemotherapy and showed no response (TRG3), showing very adverse clinical characteristics and poor survival (19). In the present study, case 1 did not respond to chemotherapy and anti-PD-1 immunotherapy. Case 2 showed TRG3 in response to neoadjuvant chemotherapy. GI NEC with SMARCA4 deficiency may have limited benefit from chemotherapy and anti-PD-1 immunotherapy. SMARCA4 in GI NEC may be a prognostic and targetable biomarker. SMARCA4-mutated cancers have a DNA repair vulnerability that can be exploited therapeutically (27). Future treatments with agents that target the epigenetic machinery, such as inhibitors against enhancer of zeste homolog 2 (EZH2) or histone deacetylase, may prove even more effective (28, 29), which might provide more therapeutic options.

## Conclusions

We retrospectively report two rare cases of GI NEC with SMARCA4 deficiency. GI NEC with SMARCA4 deficiency may not benefit from chemotherapy and has poor outcomes. SMARCA4 may be a promising targetable and prognostic biomarker for GI NEC, requiring more exploration for validation in a larger series.

## Data availability statement

The original contributions presented in the study are included in the article/supplementary material. Further inquiries can be directed to the corresponding author/s.

## Ethics statement

The studies involving humans were approved by Ethics approval was obtained from the respective ethics committees of West China Hospital, Sichuan University, China (NO.2022317). The studies were conducted in accordance with the local legislation and institutional requirements. The human samples used in this study were acquired from primarily isolated as part of your previous study for which ethical approval was obtained. Written informed consent for participation was not required from the participants or the participants’ legal guardians/next of kin in accordance with the national legislation and institutional requirements. Written informed consent was obtained from the individual(s) for the publication of any potentially identifiable images or data included in this article.

## Author contributions

PZ: Data curation, Writing – original draft, Writing – review & editing. YF: Formal Analysis, Software, Methodology. WW: Funding acquisition, Project administration, Writing – review & editing.
